# Deciphering the causal association and co-disease mechanisms between psoriasis and breast cancer

**DOI:** 10.3389/fimmu.2024.1304888

**Published:** 2024-03-28

**Authors:** Xujia Li, Lingli Huang, Yue Yan, Yuming Rong, Xuxian Chen, Mengge Gao, Jinsheng Huang

**Affiliations:** ^1^ VIP Department, Sun Yat-sen University Cancer Center, Guangzhou, China; ^2^ State Key Laboratory of Oncology in South China, Sun Yat-sen University Cancer Center, Guangzhou, China; ^3^ Collaborative Innovation Center for Cancer Medicine, Sun Yat-sen University Cancer Center, Guangzhou, China; ^4^ Guangdong Provincial Clinical Research Center for Cancer, Sun Yat-sen University Cancer Center, 651 Dongfeng Road East, Guangzhou, China; ^5^ Department of Clinical Nutrition, Huadu District People’s Hospital, Southern Medical University, Guangzhou, Guangdong, China

**Keywords:** psoriasis, breast cancer, causal effect, Mendelian randomization, co-disease mechanisms

## Abstract

**Background:**

Prior research has indicated a link between psoriasis and the susceptibility to breast cancer (BC); however, a definitive causal relationship remains elusive. This study sought to elucidate the causal connection and shared underlying mechanisms between psoriasis and BC through bidirectional Mendelian randomization (MR) and bioinformatic approaches.

**Methods:**

We employed a bidirectional MR approach to examine the potential causal connection between psoriasis and BC. Genetic data pertaining to psoriasis and BC were sourced from extensive published genome-wide association studies. The inverse -variance weighted or wald ratio served as the primary method for estimating causal effects. Sensitivity analysis of the MR results was applied with multiple methods. Leveraged datasets from the Gene Expression Omnibus and the Cancer Genome Atlas repositories to identify common differentially expressed genes, shedding light on the shared mechanisms underlying these two conditions.

**Results:**

The MR analysis revealed that when considering psoriasis as an exposure factor, the incidences of BC (OR=1.027) and estrogen receptor negative (ER-) BC (OR=1.054) were higher than in the general population. When using Her2+ BC as an exposure factor, the risk of psoriasis was 0.822 times higher (OR=0.822) than in the general population. Sensitivity analysis indicated that the results were robust. Transcriptome analysis showed that CXCL13 and CCL20 were activated in both BC and psoriasis. Both diseases were also linked to neutrophil chemotaxis, the IL-17 pathway, and the chemokine pathway.

**Conclusion:**

The results suggest that psoriasis may increase the risk of BC, especially ER- BC, while reverse MR suggests a decreased risk of psoriasis in Her2+ BC. Transcriptome analysis revealed a shared mechanism between psoriasis and BC.

## 1 Introduction

Breast cancer (BC) is one of the most prevalent malignant tumors affecting the female population (1). In the year 2020, there were approximately 2.3 million fresh BC cases reported worldwide, comprising 11.7% of the total cancer incidents (2). Psoriasis is a chronic immune-mediated disease (3), and the association between autoimmunity and cancer is well recognized (4). While the pathophysiology of psoriasis remains intricate, we have pinpointed a crucial role played by T cells. Furthermore, specific pathological processes, including the activation of the tumor necrosis factor (TNF)/interleukin (IL-)23/IL-17 cytokine axis, contribute significantly to the differentiation and activation of effector T cells, as well as their accumulation within affected tissues (5, 6). Epigenetic modifications play a significant role in the molecular pathogenesis of psoriasis, as they can modify gene expression without altering the underlying genomic sequence (7, 8). Changes in epigenetic characteristics have been evident across various autoimmune diseases (9). Despite previous research revealing an association between psoriasis and the risk of BC (10, 11), elucidating the precise role of psoriasis in the development of BC remains a formidable challenge. Further investigations are warranted to ascertain the existence of causal genetic molecular mechanisms linking these two conditions.

Genome-wide association studies (GWAS) have transformed the landscape of complex disease genetics by uncovering associations between genotypes and phenotypes through the examination of millions of genetic variations (12). GWAS is a comprehensive genomic analysis technique designed to uncover associations between common single nucleotide polymorphisms (SNPs) and various diseases or traits. It offers novel avenues for comprehending the pathogenesis of complex diseases. Mendelian Randomization (MR) analyses utilize genetic variants as instrumental variables (IVs), typically in the form of SNPs, to infer potential causality—that is, the link between an exposure factor and an outcome (13–15). Since SNPs are randomly allocated during conception and remain unaffected by confounding factors, MR analysis minimizes the influence of confounders and reverse causation. Consequently, MR analysis can provide more robust evidence compared to traditional observational studies in establishing causality (15, 16).

We undertook this European-based investigation to delve into the potential causal linkage between psoriasis and BC. This study enhances our comprehension of the involvement of psoriasis in the initiation and advancement of BC. Additionally, it lends support to the advancement of more efficient cancer surveillance initiatives for the early detection of cancer or precancerous lesions, thus mitigating the burden of care.

## 2 Methods

**Figure 1:**
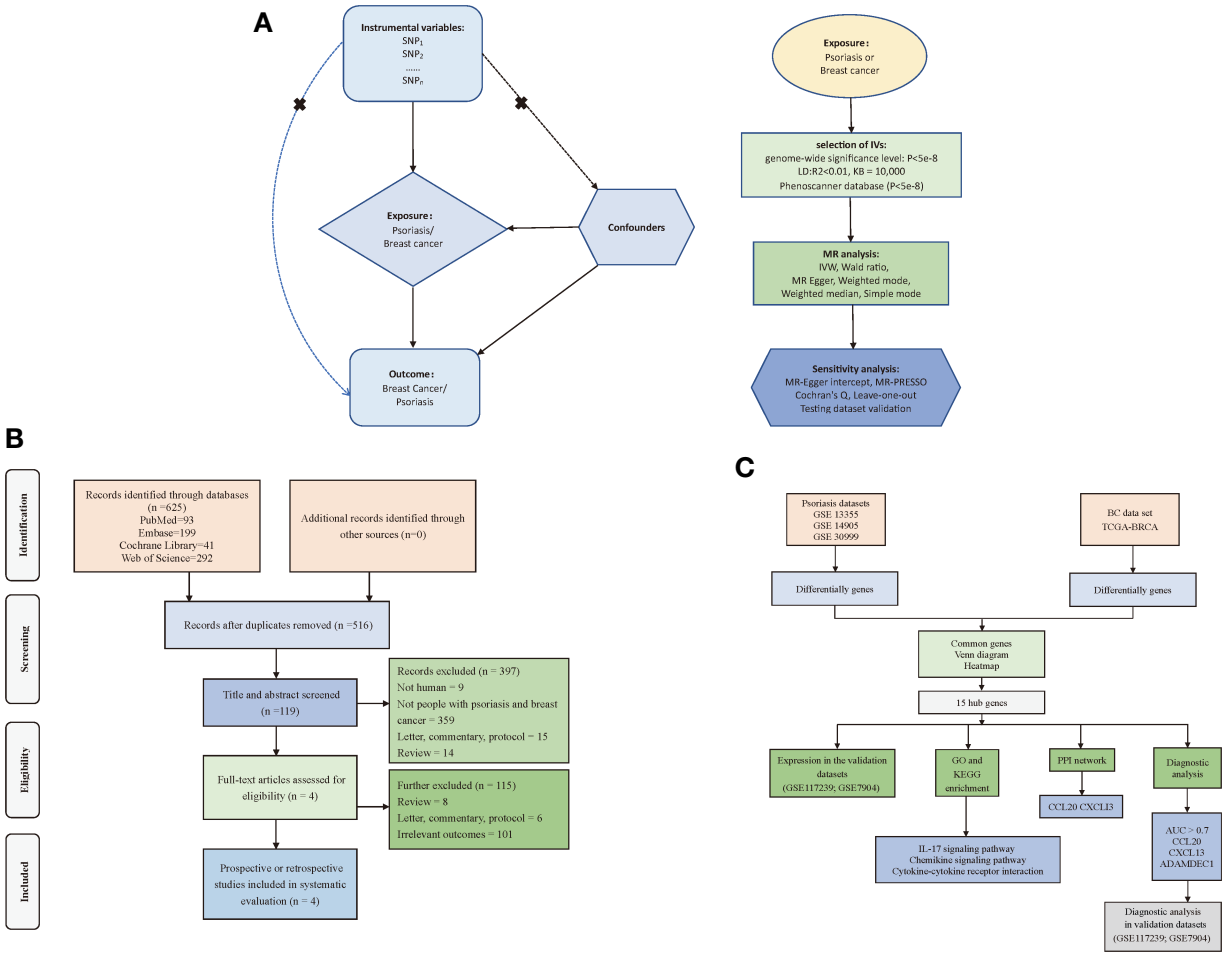
Flow of study design. **(A)** Principles and main processes of Mendelian randomization; **(B)** Literature screening process for systematic evaluation; **(C)** Transcriptome-based co-morbidity exploration process. MR, Mendelian randomization; BC, Breast cancer, IVs, Instrumental variables; SNP, Single nucleotide polymorphisms; IVW, Inverse-variance weighted; PPI, Protein-Protein Interaction; LD, Linkage disequilibrium; KB, Pairwise distance.

To investigate the causal relationship between psoriasis and BC, we employed a bidirectional two-sample MR approach. In this investigation, we employed psoriasis and BC as both exposure and outcome variables. To ensure the rigor of our study, we employed independent genetic variants as IVs, subject to meeting three critical criteria: (1) IVs were required to exhibit a robust association with the exposure; (2) no pleiotropic association of IVs with any known confounders; (3) IVs were assessed for their lack of association with outcome, except when related to exposure. We acquired genetic data for psoriasis and BC from distinct and non-overlapping GWAS datasets to ensure the independence of our analyses (**Figure 1A**). Furthermore, we systematically reviewed prior studies (**Figure 1B**) and conducted a transcriptome-based investigation into shared mechanisms (**Figure 1C**) between these two conditions. For this research, we adhered to the STROBE-MR statement for reporting MR studies (**Supplementary Table S1**) (17). Given that the datasets used in this study were available from public databases, additional ethical approval or informed consent were not required.

### 2.1 MR analysis

#### 2.1.1 Data sources

Data regarding psoriasis were sourced from FinnGen (https://www.finngen.fi) and the European Bioinformatics Institute (EBI) (https://www.ebi.ac.uk/). These databases comprised 216,752 and 33,394 individuals of European descent, respectively. Importantly, our analysis did not identify any weak IVs, and all F-statistics > 10, indicating minimal bias due to weak instruments (**Supplementary Table S2**). The FinnGen’s primary objective is to collect and scrutinize genomic and health data from 500,000 participants in the Finnish BioBank, with the aim of enhancing our comprehension of genomic influences on health (18, 19). EBI offers freely accessible bioinformatics resources to the scientific community, champions fundamental research, offers training opportunities, and disseminates state-of-the-art technologies (20). EBI is responsible for the management and upkeep of various extensive public bioinformatics databases, spanning diverse domains like genomics, proteomics, chemoinformatics, transcriptomics, and systems biology. Additionally, EBI provides a diverse array of tools to aid researchers in the analysis and sharing of information.

BC data were sourced from the Integrative Epidemiology Unit (IEU) database (IEU Open GWAS project) (https://gwas.mrcieu.ac.uk/) and the FinnGen database, both of which are associated with GWAS studies related to cancer. In reverse MR analyses, all F-statistics > 10, signifying minimal bias stemming from weak IVs (**Supplementary Table S3**). Within the IEU database, there were 89,677, 50,225, and 69,970 individuals of European descent included for BC, estrogen receptor-negative (ER-) BC, and ER-positive (ER+) BC data, respectively. As for the FinnGen database, it encompassed data from 102,359 and 103,530 European individuals for Her2- and Her2+ BC, respectively. The IEU database encompasses a wealth of information, comprising over 214.7 billion genetic associations drawn from 42,484 GWAS summary datasets (21).

Furthermore, to strengthen the credibility of the findings, we extracted data on BC and psoriasis from the UK Biobank (UKB) cohort as testing dataset. The UKB cohort is a prospective general population cohort that was assessed at baseline between 2006 and 2010 at 22 different assessment centers in the UK. A total of 502,628 participants aged between 40 and 70 years were recruited to complete questionnaires covering topics such as lifestyle, sociodemographic characteristics, physical and mental health, and habitual food intake (22, 23). Summary data for the UKB cohort were available from the IEU database. **Supplementary Table S4** displayed the characteristics of the psoriasis and BC datasets.

#### 2.1.2 Instrumental variable selection

To ensure the robustness of our conclusions regarding the mutual risk of morbidity between psoriasis and BC, we implemented rigorous quality control measures to select the most appropriate IVs. Here are the key steps we followed: (1) we identified SNPs that displayed significant associations with exposure (psoriasis or BC) across the entire genome, employing a significance threshold of P < 5 × 10^-8^; (2) we applied a minor allele frequency (MAF) threshold of 0.01 for the specific variant of interest; (3) to minimize the potential for biased results, we carefully chose SNPs strongly associated with exposure (psoriasis or BC) that demonstrated no linkage disequilibrium (LD) (*R^2^* < 0.01, and clumping distance = 10,000 kb); (4) we excluded palindromic SNPs (e.g., those with A/T or G/C alleles) to prevent any distortions in chain orientation or allele coding. In order to ensure a sufficient number of SNPs, we also tried to infer positive strand alleles, using allele frequencies for palindromes; (5) we cross-referenced these alleles with the human genome reference sequence (build 37) and removed any SNPs that were unspecified or duplicated; (6) Following the PhenoScanner database (24), these SNPs as IVs were examined whether they might be strongly associated with the occurrence of BC (estrogen, reproductive factors, and so on) or psoriasis (mechanical stress, smoking, alcohol use, and so forth).

#### 2.1.3 MR analysis and sensitivity analysis

We employed a two-sample MR study to explore the potential causal relationship between psoriasis and BC, encompassing both ER and Her2 receptor subtypes. When the number of SNPs was greater than or equal to two, we utilized the inverse-variance weighted (IVW) method as the primary analysis approach (25). In cases where only one SNP was available, we employed the Wald ratio method for analysis. Additionally, we applied supplementary methods, including weighted mode (26), MR-Egger (27), weighted median (28), and simple mode (29). In certain scenarios, the IVW method demonstrated slightly superior performance compared to the other methods (28). To reduce false positives in the findings, we corrected the results of all MR analyses for multiple testing using the method of false discovery rate (FDR). FDR < 0.05 indicated a significant causal effect, while FDR ≥ 0.05 and P < 0.05 denoted a suggestive causal effect.

To ensure the reliability of our findings, we conducted sensitivity analysis. Firstly, we employed MR-Pleiotropy Residual Sum and Outlier (PRESSO) (30) and examined the MR-Egger intercept to evaluate the potential presence of horizontal pleiotropy. The MR-PRESSO method allowed us to identify and exclude SNPs that might introduce bias. P ≥ 0.05 indicated the absence of horizontal pleiotropy, allowing us to include the remaining SNPs in the MR analysis. A deviation of the MR-Egger intercept from the origin signaled potential horizontal pleiotropy of the IVs (P < 0.05), while no evidence of horizontal pleiotropy was indicated when P ≥ 0.05. Secondly, as a supplementary sensitivity analysis, we employed the weighted median method to assess the robustness of the MR estimates. Thirdly, we calculated the F-statistic to gauge the potential bias stemming from weak instrumental variables. F-statistic < 10 suggested a weak IV, which could introduce bias and therefore needed to be excluded (31). Additionally, we utilized Cochran’s Q statistic to detect heterogeneity within the IVW models. If the Q-value exceeded the number of instruments minus one, it suggested the presence of heterogeneity and ineffective instruments. P < 0.05 indicated the potential existence of heterogeneity (32). Lastly, all results that appeared statistically significant were validated using the testing dataset to investigate the robustness of the MR analysis as well as to reduce possible potential false-negative errors after FDR correction.

All these analyses were conducted using the open-source statistical software R (version 4.3.0) along with the TwoSampleMR package (version 0.5.7).

#### 2.1.4 Reverse-direction MR analysis

We explored the potential causal relationship between BC and psoriasis through reverse-direction MR analysis. In our analysis, when only one SNP was identified, we employed the wald ratio method for this single-SNP analysis. Given the limited availability of only one SNP for analysis, we were unable to conduct assessments for heterogeneity, pleiotropy, and sensitivity. Furthermore, when there were multiple SNPs characterized, we utilized five MR methods including IVW, weighted mode, MR-Egger, weighted median, and simple mode. The sensitivity analysis was conducted in the same way as the forward MR.

### 2.2 Systematic evaluation approach

We conducted a comprehensive search across four databases (Embase, PubMed, Cochrane Library, and Web of Science) to identify studies investigating the association between psoriasis and BC. Our search included keywords such as psoriasis, BC, and disease risk. The search period extended from the inception of the database to September 2023. Inclusion criteria were as follows: (1) prospective or retrospective studies, (2) confirmed diagnoses of both psoriasis and BC, (3) studies examining the risk of BC in psoriasis patients, (4) availability of complete primary data suitable for direct or indirect statistical analysis, and (5) publication of articles in the English language. Exclusion criteria encompassed: (1) diagnosis of malignancy prior to psoriasis diagnosis, (2) studies lacking sufficient published data for analysis, and (3) articles falling into categories such as meta-analyses, letters, commentaries, editorial materials, case reports, or expert opinions. Ultimately, four studies meeting these criteria were identified and subjected to systematic evaluation. The evaluation encompassed new BC cases, incidence rates per 100,000 person-years (PY), hazard ratios (HR), odds ratios (OR), and standardized incidence rates (SIR).

### 2.3 Transcriptome analysis

#### 2.3.1 Data acquisition and processing

We obtained the datasets for psoriasis and BC from the Gene Expression Omnibus (GEO) and the Cancer Genome Atlas (TCGA). Differential expression analysis was conducted using the limma package. In this analysis, we set a log2Foldchange threshold of 0.5 for the three psoriasis datasets (GSE13355, GSE14905, and GSE30999), and a log2Foldchange threshold of 1.5 for the TCGA- BRCA dataset of BC. All adjusted P < 0.05 were considered statistically different. Differentially expressed genes (DEGs) were identified separately for each dataset, and the DEGs obtained were then taken to be intersected. The results of this intersection analysis were visualized using upset plots and Venn diagrams. To validate the findings, we further examined the DEGs in both the GEO and TCGA datasets. **Supplementary Table S5** provided details on the characteristics of the transcriptome dataset.

#### 2.3.2 Functional enrichment analysis

Gene ontology (GO) and The Kyoto Encyclopedia of Genes and Genomes (KEGG) were used to further explore relevant biological pathways and higher genomic functions. We used the clusterProfiler and org.Hs.eg.db packages to enrich the KEGG pathway and evaluate the GO functionality of possible targets. The threshold was set to P < 0.01.

#### 2.3.3 Protein-protein interaction network analysis

Potential protein interactions with DEGs were collected and integrated by using the STRING database (https://string-db.org/). The obtained genes were analyzed by PPI network analysis. Cytoscape was used for further subsequent analysis and visualization. The top 3 genes related signaling pathways were reconstructed through the CytoHubba and MCODE.

#### 2.3.4 Receiver operator characteristic curve

ROC curves were performed by using the pROC package (v1.17.0.1) and plotted with the ggplot2 package. Area under the curve (AUC) more than 0.7 has the good diagnosis accuracy.

## 3 Results

### 3.1 MR analysis

#### 3.1.1 Genetic instruments

Following a rigorous quality control process, we selected a total of 146 genetic variants (SNPs) associated with the exposure phenotype for the psoriasis validation analysis, with a significance threshold set at P < 5 × 10^-8^ (**Supplementary Table S2**). For the reverse MR analysis focused on BC, 87 genetic variants (SNPs) linked to the exposure phenotype were used (**Supplementary Table S3**). The specific count of selected IVs and the screening process were provided in **Supplementary Tables S2**, **S3**.

#### 3.1.2 Causal relationship between psoriasis and BC

**Figure 2:**
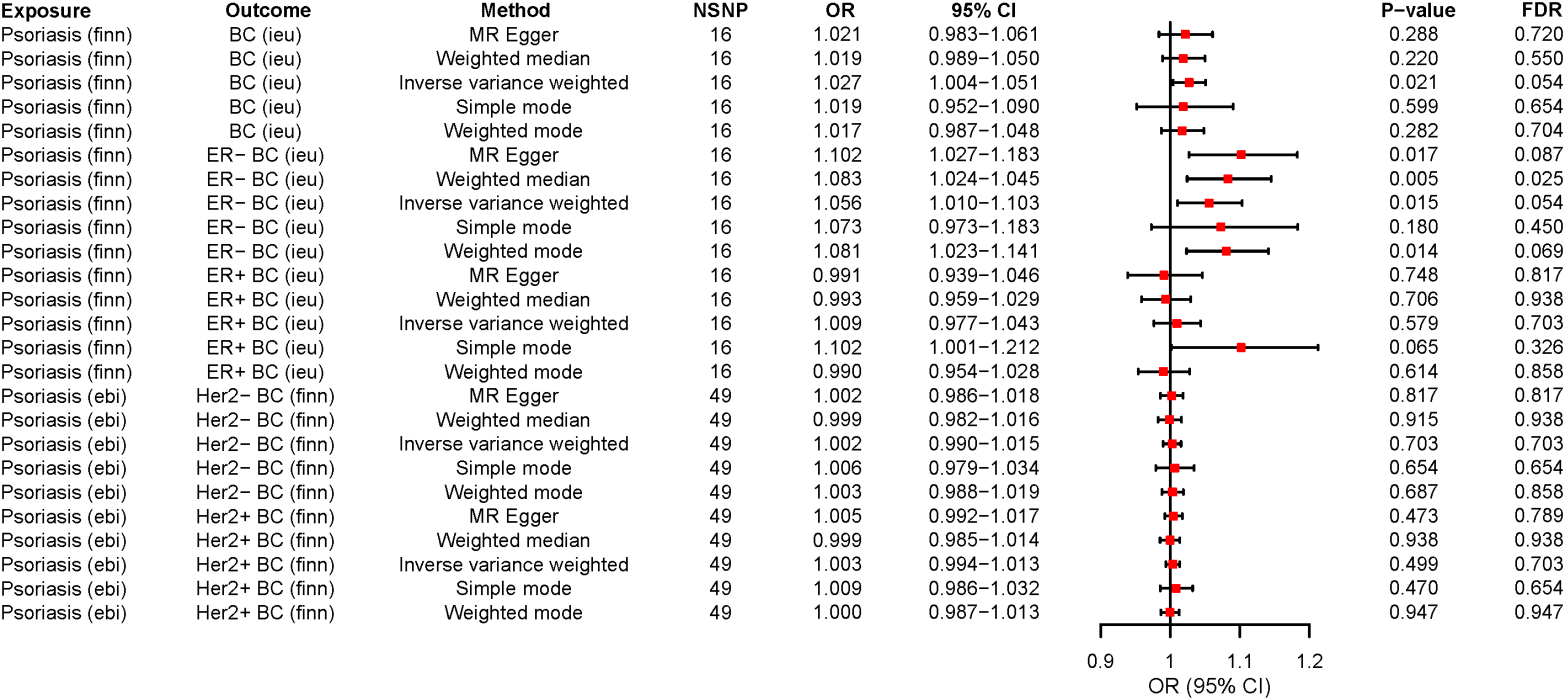
The forest plots for psoriasis on breast cancer risk. OR>1 indicates the positive correlation between psoriasis and a particular breast cancer. OR<1 indicates the Negative correlation between the two. The P < 0.05 indicated statistical significance. MR, Mendelian randomization; ER-, ER-negative; ER+, ER-positive; SNP, Single nucleotide polymorphism; OR, Odds ratio; 95%CI, 95% confidence interval; NSNP, Number of SNP; FDR, False discovery rate; BC, Breast cancer.

**Figure 3:**
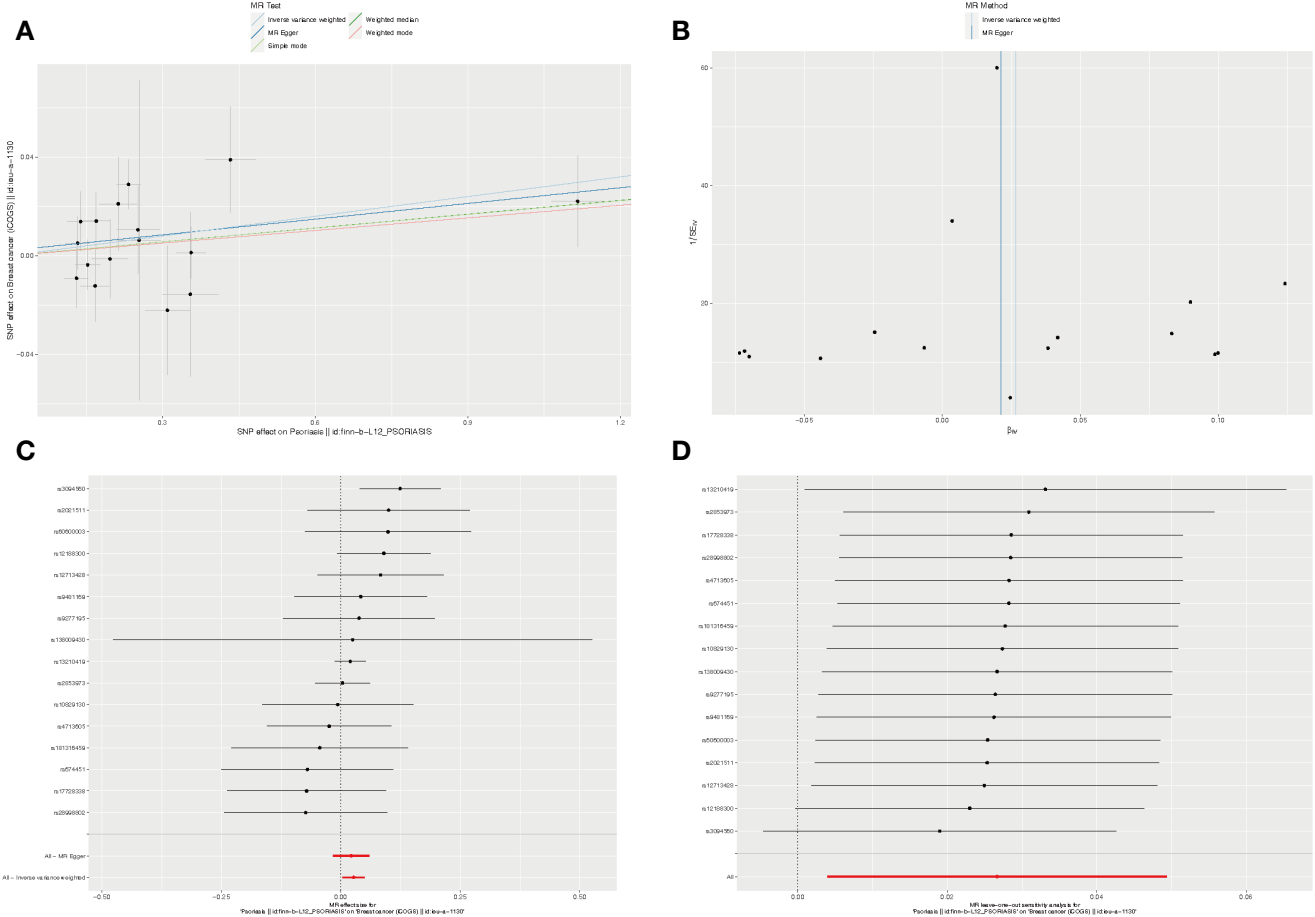
The causality of psoriasis on breast cancer risk in European populations. **(A)** Scatter plot. The slope of each line denotes the estimated effect of per mendelian randomization method. **(B)** Funnel plot. Vertical lines represent estimates with all SNPs. **(C)** Forest plot. The red points demonstrate the integrated estimates using all SNPs together, using IVW method. Horizontal lines represent 95% confidence intervals. **(D)** Leave-one-out analysis. Black points depict the IVW method was used to assess the causal effect, excluding single specific variant from the analysis. The red point denotes the inverse-variance weighted estimate using all SNPs. MR, Mendelian randomization; SNPs, Single nucleotide polymorphisms; IVW, Inverse-variance weighted.

In our MR study, we observed that while the application of various analytical methods, including MR-Egger, weighted mode, simple mode, and weighted median, did not reveal a significant correlation between psoriasis and BC (P > 0.05), the analysis employing the IVW approach indicated that psoriasis indeed represents a risk factor for BC development and there was a causal relationship between them. These findings established that psoriasis (OR=1.027, 95% CI: 1.003-1.050, P=0.021, FDR=0.054) is approximately 1.027 times more likely to predispose individuals to BC compared to the general population when considered as an exposure factor (**Supplementary Table S6**; **Figures 2**, **3**). Meanwhile, the IVW results suggested that compared to the normal population, psoriasis was associated with a 1.056-fold increased risk of developing ER- BC (OR=1.056, 95% CI: 1.010-1.103, P=0.015, FDR=0.054), whereas there was no statistically significant difference in the risk of developing ER+ BC (P > 0.05; **Supplementary Table S6**; **Figures 2**; **S1**, **S2**). The results of the remaining four MR analysis methods were in general agreement with the IVW results. In addition, no causal relationship was observed between psoriasis and the Her2 receptor subtype of BC (**Supplementary Figures S3**, **S4**).

Remarkably, when we conducted a reverse MR analysis, an unexpected revelation emerged. It came to light that the Her2+ BC actually functions as a protective factor against psoriasis and there was a causal relationship between them. The wald ratio analysis demonstrated that employing the Her2+ BC subtype as an exposure factor (OR=0.822, 95% CI: 0.679-0.995, P=0.044, FDR=0.059) results in a 0.822-fold reduction in the risk of psoriasis development when compared to the general population (**Supplementary Table S7**; **Supplementary Figure S5**). When BC and its other subtypes (ER-, ER+, and Her2-) were investigated as exposures, we did not identify the causal relationship between these exposures and psoriasis risk (**Supplementary Table S7**; **Supplementary Figures S5**–**S7**).

#### 3.1.3 Sensitivity analysis

To ensure the robustness of our MR causal effect estimates, we conducted multiple sensitivity analysis. Cochran’s Q test indicated that all of our results were devoid of bias and showed no signs of heterogeneous associations (**Supplementary Table S8**). Additionally, a pleiotropy test employing both the MR-Egger intercept and MR-PRESSO methods yielded all P > 0.05, signifying an absence of pleiotropy (**Supplementary Table S8**). Furthermore, sensitivity analysis suggested that the results were no heterogeneity or pleiotropy when BC and ER subtype of BC were utilized as exposures for reverse MR (**Supplementary Table S9**). However, during the reverse MR analysis, only a single SNP was identified when utilizing the Her2 receptor subtype of BC as the exposure variable. Consequently, we were unable to perform heterogeneity test or pleiotropy test in this context (**Supplementary Table S9**).

Furthermore, we employed the leave-one-out method for sensitivity analysis. After individually excluding each SNP, our findings revealed that no single SNP exerted a significant influence on the overall causal estimates derived from all instrumental variables. This reaffirmed the reliability of the results obtained in our MR study (**Supplementary Tables S10**, **S11**).

Finally, to further confirm the causal relationship between psoriasis and breast cancer, we performed the MR analysis again for statistically significant exposures and outcomes using the testing dataset. In the testing dataset, the IVW results indicated that psoriasis remained a risk factor contributing to an elevated risk of BC development (OR=1.001, 95% CI: 1.000-1.003, P=0.032), whereas there was no causal association between Her2+ BC and psoriasis risk (OR=1.000, 95% CI: 0.999-1.001, P=0.907; **Supplementary Figure S9**). The results of the other four methods were generally consistent with the IVW results. However, due to the unavailability of the testing dataset for ER- BC, the causal relationship between psoriasis and ER- BC was unable to validate.

### 3.2 Systematic evaluation

Our study also included a comprehensive evaluation of pertinent literature through a systematic review. Among the studies examined, Boffetta P et al. (33) (SIR=1.27, 95% CI: 1.00-1.58) and Kimball AB et al. (34) (OR=1.32, 95% CI: 1.24-1.40) indicated that psoriasis serves as a risk factor for BC. Conversely, studies conducted by Chiesa Fuxench ZC et al. (35).

(HR=1.04, 95% CI: 0.97-1.12) and Prizment AE et al. (36) (HR=1.00, 95% CI: 0.70-1.50) suggested that there was no significant difference in BC incidence between patients with psoriasis and the general population (**Table 1**).

**Table 1:** Systematic evaluation of the risk of developing psoriasis in relation to breast cancer.

Study	Data Sources	Participants, No.			New breast cancer cases, No.		Incidence per 100 000 PY		Observation indicators
		Control	Psoriasis	Total sample size	Control	Psoriasis	Control	Psoriasis	Psoriasis vs Control
Chiesa Fuxench ZC	The Health Improvement Network (THIN)	937716	198366	1136082	6281	1118	180.87	177.84	HR: 1.04 (0.97-1.12)
Prizment AE	IWHS/Medicare data/Lowa SEER cancer registry	402199	5477	407676	2037	29	510	530	HR: 1.00 (0.70–1.50)
Boffetta P	Swedish Cancer Register	84003	9773	93776	NA	78	NA	NA	SIR: 1.27(1.00-1.58)
Kimball AB	Truven Health Analytics Marketscan Database	179066	179066	358132	NA	NA	NA	NA	OR: 1.32(1.24-1.40)

OR, Odds ratio; HR, Hazard ratios; SIR, Standardized incidence rates; NA, Not applicable; PY, Person-years; No, Number.

### 3.3 Transcriptome-based exploration of co-morbid mechanisms

#### 3.3.1 CXCL13 and CCL20 activation in psoriasis and BC

**Figure 4:**
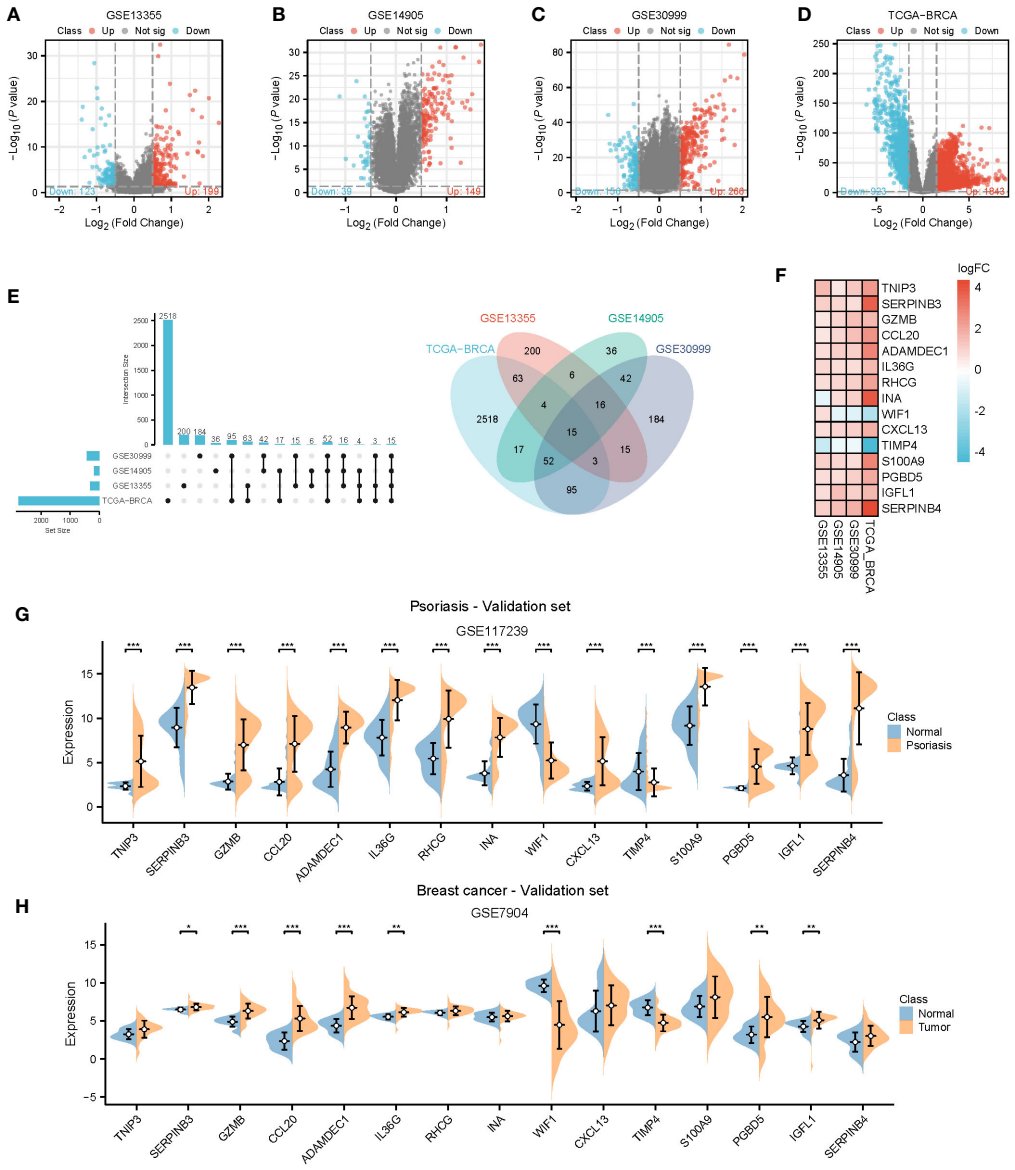
Identification of common genes. **(A–D)** Volcano plots for differentially genes (DEGs) expression. Red dots represent upregulated gene expression, whereas blue dots represent downregulated ones, and the grey dots stand for no significant difference. **(A–C)** Psoriasis dataset; **(D)** Breast cancer dataset. **(E, F)** Venn diagram and heatmap of genes common to both psoriasis and breast cancer. **(G, H)** Expression of 15 common genes in the validation datasets. TCGA, The Cancer Genome Atlas; DEGs, Differentially genes; *, Statistically significant. Wilcoxon test. *p<0.05; **p<0.01; ***p<0.001.

To provide further clarity regarding the association between psoriasis and BC, we selected three GEO datasets related to psoriasis and performed screening with a log2Foldchange threshold of 0.5. Subsequently, we conducted differential expression analysis using the limma package. In the GSE13355 dataset, we identified 199 up-regulated genes and 123 down-regulated genes (**Figure 4A**). In the GSE14905 dataset, we found 149 up-regulated genes and 39 down-regulated genes (**Figure 4B**). In the GSE30999 dataset, 266 up-regulated genes and 156 down-regulated genes were detected (**Figure 4C**). For BC, we identified 1843 up-regulated genes and 923 down-regulated genes using the TCGA database with a log2Foldchange of 1.5 (**Figure 4D**). By intersecting the differential genes identified in all four datasets, we identified 15 common genes (**Figure 4E**) that exhibited high expression in both BC and psoriasis, with the exception of INA, WIF1, and TIMP4. We validated the expression of these 15 genes in the psoriasis dataset GSE117239 and the BC dataset GSE7904. All of these genes were confirmed in both datasets, except for TNIP3, RHCG, INA, S100A9, and SERPINB4, which did not show statistically significant differences in BC (**Figures 4F–H**).

**Figure 5:**
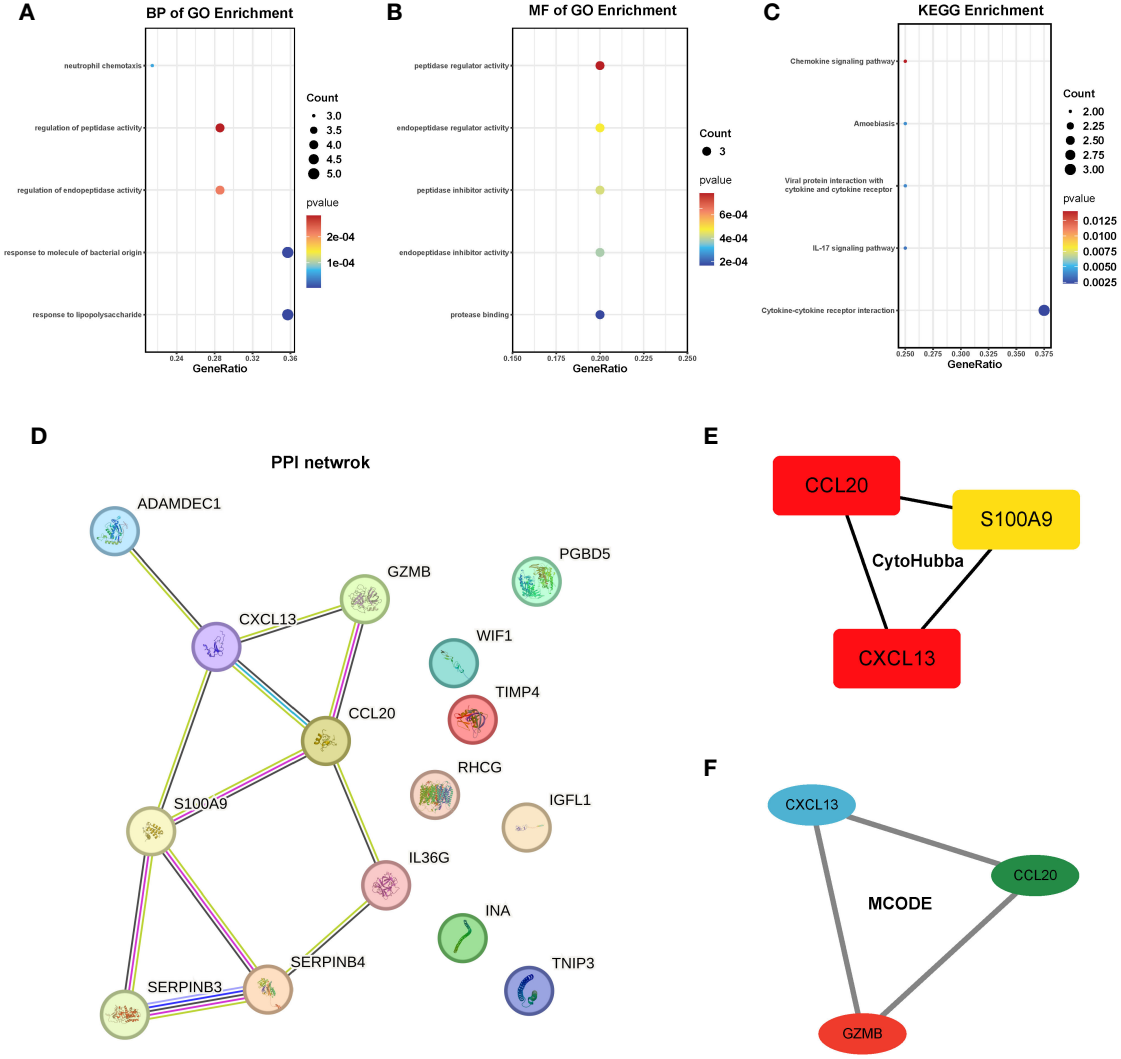
Enrichment analysis and PPI network of the 15 hub genes. **(A-C)** GO and KEGG pathway analysis. **(D)** PPI network. **(E, F)** The top 3 hub genes were identified by the CytoHubba and MCODE. GO, Gene ontology; KEGG, The Kyoto Encyclopedia of Genes and Genomes.

To gain deeper insights into the pathways influenced by these common genes, we conducted enrichment analyses using GO and KEGG. The results revealed significant enrichments in biological processes (BP) and molecular functions (MF) related to neutrophil chemotaxis and peptidase activation. In the KEGG analysis, pathways related to chemokines and IL-17 were prominently enriched. However, due to the limited number of genes, the cellular component (CC) category did not yield any meaningful pathway enrichments (**Figures 5A–C**). To further explore the interactions among these genes, we constructed a protein-protein interaction (PPI) network using the String database, which revealed 15 hub genes (**Figure 5D**). Employing CytoHubba and MCODE algorithms, we identified the top three hub genes as CCL20, CXCL13, and S100A9, along with another set of top hub genes: CCL20, CXCL13, and GZMB (**Figures 5D–F**).

#### 3.3.2 Diagnostic efficacy and prognostic evaluation of 15 Hub Genes

**Figure 6:**
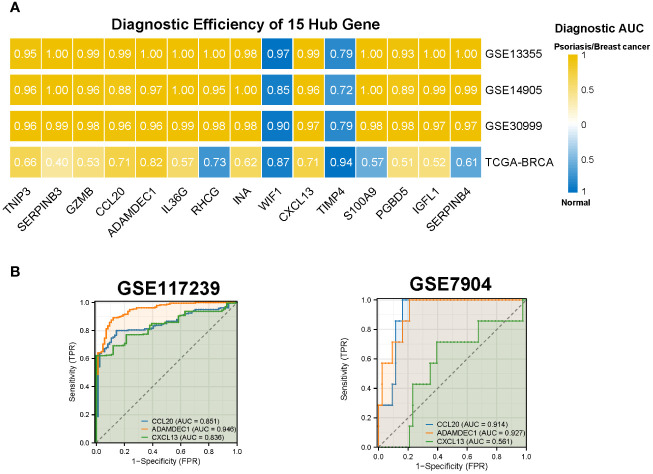
Diagnostic efficiency of the 15 hub genes. **(A)** Diagnostic AUC of 15 hub genes in psoriasis and breast cancer datasets. The AUC greater than 0.7 was considered to have high accuracy. **(B)** The AUC value of CCL20, ADAMDEC1, CXCL13 in the validation datasets. AUC, Area under curve; TCGA, The Cancer Genome Atlas.

We assessed the diagnostic efficacy of the 15 hub genes using the pROC package, considering an AUC greater than 0.7 as indicative of good diagnostic performance. The results demonstrated that CCL20, ADAMDEC1, and CXCL13 exhibited strong diagnostic performance in both BC and psoriasis. These findings were further validated in the psoriasis dataset GSE117239 and the BC dataset GSE7904 (**Figures 6A, B**).

## 4 Discussion

Psoriasis, characterized as an autoimmune disease (37, 38), is closely intertwined with autoimmune deficiencies, which contribute significantly to cancer development and progression. Epidemiological investigations have unveiled a robust link between psoriasis and cancer. For instance, an Italian population-based retrospective study conducted by Borghi A et al. revealed that individuals afflicted with plaque psoriasis exhibited an elevated cancer risk (SIR = 1.30, CI 95%: 0.9-1.8). Similarly, a study conducted by Calapai F et al. underscored an augmented cancer risk in psoriasis patients compared to the general population. The pathogenesis of psoriasis involves immune system activation, intricate interplay between T cells, dendritic cells, and cytokines, including IL-12, IL-17, and IL-23. The equilibrium between IL-12 and IL-23 assumes paramount importance in carcinogenesis, and disruptions in IL-12 and/or IL-23 signaling can potentiate tumor growth. Extensive genetic association studies have also pinpointed the correlation between single nucleotide polymorphisms in IL-23 and an escalated risk of malignancy (39). Furthermore, research conducted by Ahmed AR et al. unveiled the presence of autoantibodies to laminin-332 (LM-332Pg) across various autoimmune diseases, including psoriasis, with a concomitant heightened cancer risk. Nevertheless, the question of whether psoriasis is associated with an increased risk of developing BC remains elusive. Hence, we undertook this study with the objective of exploring the causal connection between psoriasis and BC, along with investigating the underlying mechanisms of these co-morbid conditions using transcriptomic approaches.

Our study chosen genetic variants from psoriasis GWAS that exhibited strong associations and comprehensive genetic data. With this study, we found that psoriasis may be a potential risk factor for BC and established a causal relationship between them. The implications of these findings could be significant for public health interventions aimed at mitigating the risk of cancer.

Our MR analysis results revealed a notable association between Psoriasis as an exposure factor and BC (OR=1.027, 95% CI: 1.003-1.050, P=0.021), indicating that individuals with Psoriasis are approximately 1.027 times more likely to develop BC compared to the general population. These results align with the observations made by Schairer C et al. (11), who similarly reported an elevated risk of BC among individuals with psoriasis (OR=1.16, 95% CI: 1.06-1.27). Although the results of MR analysis tended to lose statistical differences after correction (FDR=0.054), psoriasis was still found to be a risk factor for BC development in the MR-PRESSO analysis (P=0.035) and testing cohort (P=0.032), suggesting that our finding was robust and reliable. In our study, we also conducted a systematic evaluation of pertinent research through an extensive literature search. Among the four studies included, it was observed that two studies (33, 34) provided evidence supporting the notion that psoriasis could be a risk factor for BC. In contrast, the remaining two studies (35, 36) concluded that there was no substantial difference in the incidence of BC between individuals with psoriasis and the general population. While it is well-documented that many autoimmune diseases have a higher prevalence among women, possibly linked to hormonal factors (40), our study did not identify an association between psoriasis and the ER+ status in BC, despite the strong association between BC and hormone levels. A study conducted by Gadalla SM et al. arrived at a similar conclusion, indicating that the risk of BC in individuals with autoimmune diseases is independent of ER status. Interestingly, our study found that psoriasis led to the higher ER- BC risk (OR=1.056, 95% CI: 1.010-1.103, P=0.015), which at the same time might have contributed to the elevated overall BC risk. Moreover, our study also revealed that psoriasis was not causally linked to the risk of BC with different Her2 receptor status. Schairer et al. demonstrated that psoriasis caused an elevated risk of progesterone receptor (PR)-positive BC, but not PR-negative BC (11). Since in the current BC cohort we only have access to a single ER status or Her2 status, it was not possible to identify BC subtypes with a diverse combination of PR or other receptors added. Therefore, the conclusions that psoriasis caused changes in the risk of single-receptor BC subtypes were not yet comprehensive. Importantly, the different BC subtypes combined from ER, PR, and Her2 receptors need to be further refined in larger populations so that more accurate findings can be drawn.

The forward MR has shown that psoriasis was the risk factor for the development of BC. Meanwhile, a Swedish population-based cohort study conducted by Yang H et al. (41) identified Her2+ BC as a risk factor for psoriasis. However, in a surprising twist, our inverse MR analysis revealed a protective association between Her2+ BC subtypes and psoriasis, marking a novel and unexpected discovery. Kim et al. have reported the development of new-onset psoriasis in a BC patient treated with trastuzumab (Her2 inhibitor) (42). Although the role of Her2 in the skin is unclear, some evidence pointed to an active role for Her2 in keratinocyte differentiation (43). Moreover, De potter et al. demonstrated that in a subpopulation of differentiated keratinocytes, some Her ligands activated a signaling pathway of Her2 heterodimers, but not of epidermal growth factor (EGFR) homodimers (43). Thus, certain Her2 inhibitors might induce alterations in normal epidermal differentiation and turnover regardless of EGFR. This to some extent implied that Her2+ BC might have the ability to reduce the risk of psoriasis. Nevertheless, the causal association between Her2+ BC and psoriasis was not validated once again in our testing dataset. Consequently, although there appeared to be a conflict with the findings of our forward MR, this conclusion needs to be viewed and analyzed with caution.

Our study delved into the shared mechanisms underlying these two conditions using transcriptome analysis. We discovered that CXCL13 and CCR20 activation was evident in both BC and psoriasis. Notably, these two genes, along with ADAMDEC1, demonstrated good diagnostic efficacy for both diseases. Further exploration through GO and KEGG enrichment analysis unveiled associations with neutrophil chemotaxis, the IL-17 pathway, and the chemokine pathway in both conditions. Of particular importance, IL-17A emerged as a central cytokine in the pathogenesis of psoriasis, driving the proliferation of epidermal keratinocytes (44). These keratinocytes, in turn, produce a multitude of antimicrobial peptides and chemokines, including CXCL1, CXCL2, CXCL8, and CCL20 (45). CCL20, in particular, plays a pivotal role in psoriasis by binding to its receptor CCR6. Moreover, CCL20 has been implicated in the progression of various cancers, such as liver, colon, breast, pancreatic, and gastric cancers (46). In summary, our transcriptome results offer insights into the potential interaction between psoriasis and BC.

Our study carries significant implications for pre-cancer screening and intervention efforts. While substantial progress has been achieved in elucidating genetic variations associated with human diseases, the majority of genetic risk factors remain enigmatic. Additional biological investigations are warranted to unveil the intricate interplay between psoriasis and BC.

Human behavior and the environments in which it manifests are intricate, shaped by the interplay between genetic factors and environmental influences (47–50). To mitigate confounding factors inherent in epidemiological investigations, we employed MR techniques. The SNPs utilized in our study exhibited robust associations with psoriasis and were subsequently cross-referenced with the BC database. The outcomes of our sensitivity analyses demonstrated statistical robustness, revealing the absence of pleiotropy or heterogeneity. Nonetheless, our investigation does have certain limitations. Firstly, it’s important to acknowledge that the key assumptions of MR analysis come with inherent limitations. Despite our efforts to minimize confounding variables, we cannot completely rule out the presence of other confounders or potential multiplicative effects. Secondly, the use of aggregated data from GWAS introduces the possibility of results being influenced by variations in quality control and selection criteria across different studies. Thirdly, it’s important to note that while MR can infer potential causal relationships, it does not provide insights into specific biological pathways. Fourth, our study primarily focused on European populations, which may limit its generalizability to other ethnic groups. Finally, the limited number of SNPs obtained in the analysis of Her2+ BC as a protective factor against psoriasis prevented us from conducting comprehensive heterogeneity, pleiotropy, and sensitivity analyses, potentially impacting the robustness of our conclusions. Furthermore, while our study did not identify an association between psoriasis and the status of ER and Her2 receptor in BC, it’s important to acknowledge that the conclusions may be influenced by the limited sample size. We look forward to the availability of more SNP data in the future, which will provide opportunities for further validation and refinement of our conclusions.

## 5 Conclusion

Our study conducted a comprehensive evaluation of the relationship between psoriasis and BC, and we have reached the following conclusions. When psoriasis was considered as an exposure factor, it might pose a risk for the development of BC, especially ER- BC. In addition, when Her2+ BC considered as an exposure factor, it might exhibit a protective effect against psoriasis. A causal relationship appeared to exist between the above-mentioned findings. Transcriptome analysis further suggested a co-morbid mechanism for psoriasis and BC. In summary, this research offers novel insights into the genetic correlations and underlying mechanisms between psoriasis and BC.

## Data availability statement

The datasets presented in this study can be found in online repositories. The names of the repository/repositories and accession number(s) can be found in the article/**Supplementary Material**.

## Author contributions

XL: Conceptualization, Data curation, Formal analysis, Software, Visualization, Writing – review & editing. LH: Conceptualization, Investigation, Methodology, Supervision, Validation, Writing – review & editing. YY: Formal analysis, Project administration, Resources, Validation, Visualization, Writing – review & editing. YR: Conceptualization, Data curation, Methodology, Software, Writing – review & editing. XC: Formal analysis, Project administration, Supervision, Validation, Writing – review & editing. MG: Investigation, Methodology, Project administration, Software, Visualization, Writing – review & editing. JH: Conceptualization, Data curation, Formal analysis, Funding acquisition, Investigation, Methodology, Project administration, Resources, Software, Supervision, Validation, Visualization, Writing – original draft, Writing – review & editing.

## Supplementary material

supplementary figure s1 The causality of psoriasis on ER negative breast cancer risk in European populations.

supplementary figure s2 The causality of psoriasis on ER positive breast cancer risk in European populations.

supplementary figure s3 The causality of psoriasis on Her-2 negative breast cancer risk in European populations.

supplementary figure s4 The causality of psoriasis on Her-2 positive breast cancer risk in European populations.

supplementary figure s5 The forest plots for breast cancer on psoriasis risk.

supplementary figure s6 The causality of breast cancer on psoriasis risk in European populations.

supplementary figure s7 The causality of ER negative breast cancer on psoriasis risk in European populations.

supplementary figure s8 The causality of ER positive breast cancer on psoriasis risk in European populations.

supplementary figure s9 Validation of statistically significant MR analysis results using the testing dataset.

supplementary table s1 STROBE-MR checklist of recommended items to address in reports of Mendelian randomization studies.

supplementary table s2 IVs associated with psoriasis (exposure) and breast cancer (outcome).

supplementary table s3 IVs associated with breast cancer (exposure) and psoriasis (outcome).

supplementary table s4 Characterization of datasets for psoriasis and breast cancer.

supplementary table s5 Characterization of datasets for transcriptome.

supplementary table s6 MR estimates of the casual relationships between psoriasis (exposure) and breast cancer (outcome) risks.

supplementary table s7 MR estimates of the casual relationships between breast cancer (exposure) and psoriasis (outcome) risks.

supplementary table s8 Sensitivity results of MR analysis between psoriasis (exposure) and breast cancer (outcome)

supplementary table s9 Sensitivity results of MR analysis between breast cancer (exposure) and psoriasis (outcome).

supplementary table s10 Sensitivity results of MR analysis between psoriasis (exposure) and breast cancer (outcome) (leave-one-out).

supplementary table s11 Sensitivity results of MR analysis between breast cancer (exposure) and psoriasis (outcome) (leave-one-out).
