# Supplementary material for: Deciphering the causal association and co-disease mechanisms between psoriasis and breast cancer
Source: Front Immunol. 2024 Mar 28;15:1304888. doi: 10.3389/fimmu.2024.1304888 (PMC11007022; doi:10.3389/fimmu.2024.1304888)
Supplement: Supplementary file 3 [file DataSheet_3.docx]

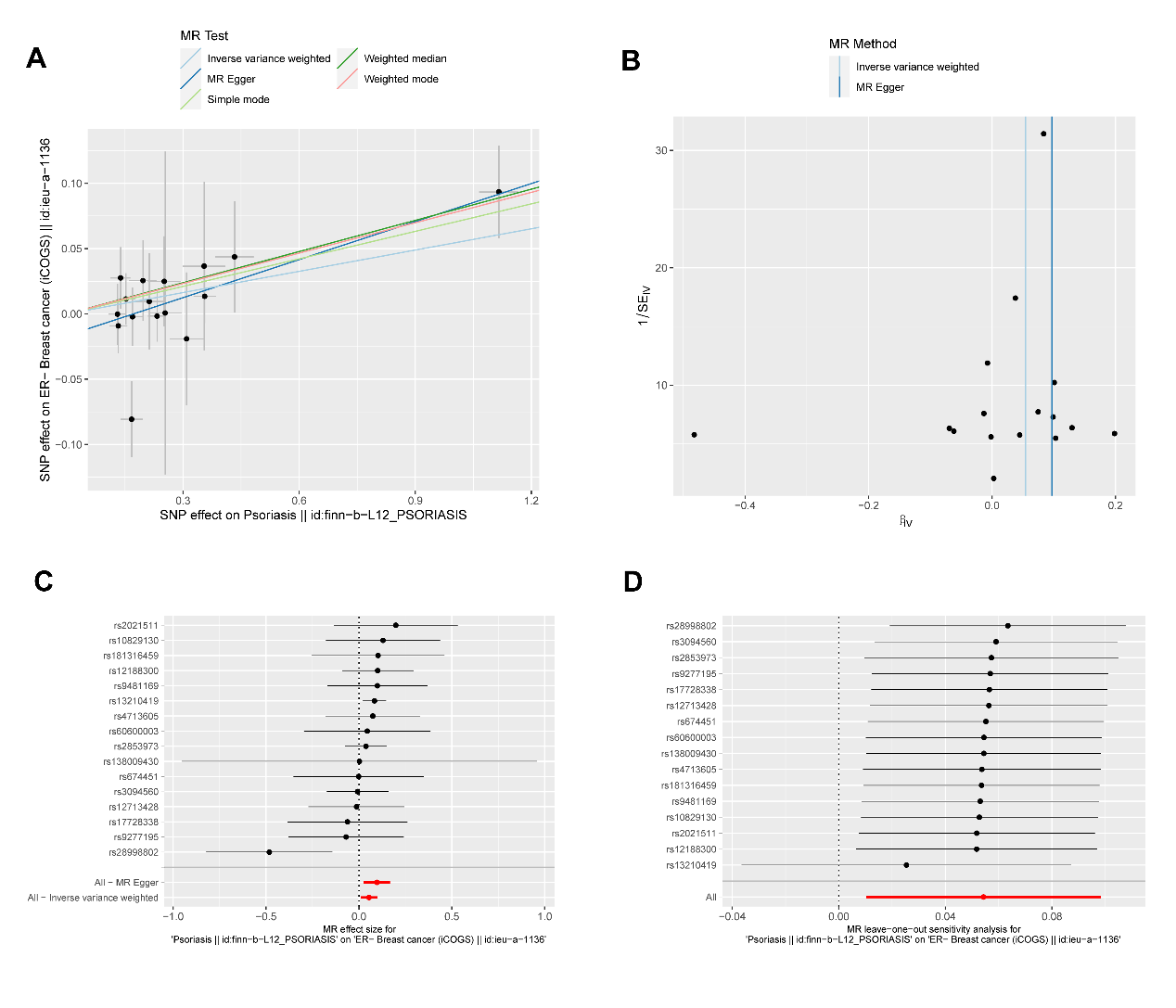


**Figure S1 The causality of psoriasis on ER negative breast cancer risk in European populations.** (A) Scatter plot. The slope of each line denotes the estimated effect of per mendelian randomization method. (B) Funnel plot. Vertical lines represent estimates with all SNPs. (C) Forest plot. The red points demonstrate the integrated estimates using all SNPs together, using IVW method. Horizontal lines represent 95% confidence intervals. (D) Leave-one-out analysis. Black points depict the IVW method was used to assess the causal effect, excluding single specific variant from the analysis. The red point denotes the inverse-variance weighted estimate using all SNPs.

**Abbreviations:** MR, Mendelian randomization; SNPs, Single nucleotide polymorphisms; IVW, Inverse-variance weighted.


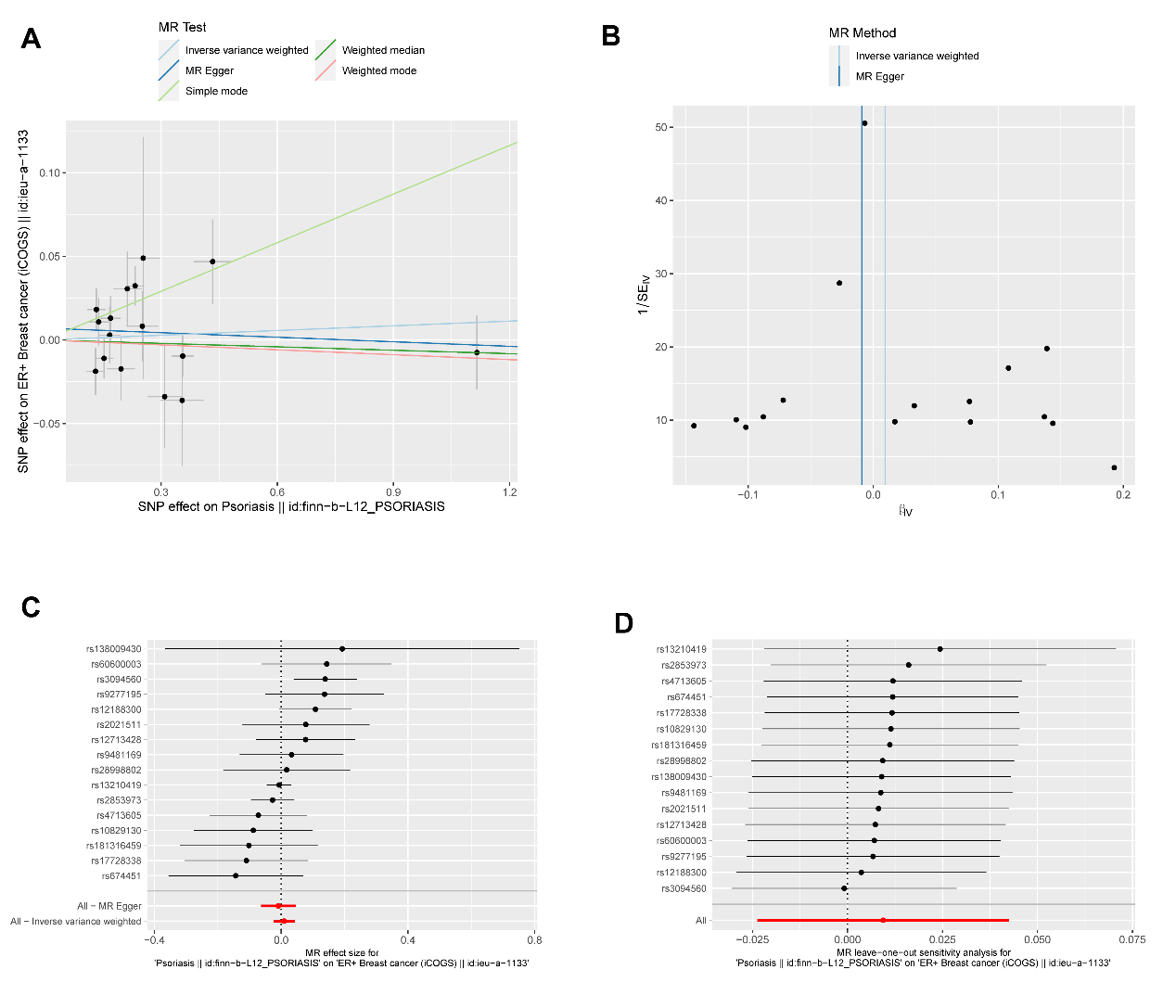


**Figure S2 The causality of psoriasis on ER positive breast cancer risk in European populations.** (A) Scatter plot. The slope of each line denotes the estimated effect of per mendelian randomization method. (B) Funnel plot. Vertical lines represent estimates with all SNPs. (C) Forest plot. The red points demonstrate the integrated estimates using all SNPs together, using IVW method. Horizontal lines represent 95% confidence intervals. (D) Leave-one-out analysis. Black points depict the IVW method was used to assess the causal effect, excluding single specific variant from the analysis. The red point denotes the inverse-variance weighted estimate using all SNPs.

**Abbreviations:** MR, Mendelian randomization; SNPs, Single nucleotide polymorphisms; IVW, Inverse-variance weighted.

**Figure S3 The causality of psoriasis on Her-2 negative breast cancer risk in European populations.** (A) Scatter plot. The slope of each line denotes the estimated effect of per mendelian randomization method. (B) Funnel plot. Vertical lines represent estimates with all SNPs. (C) Forest plot. The red points demonstrate the integrated estimates using all SNPs together, using IVW method. Horizontal lines represent 95% confidence intervals. (D) Leave-one-out analysis. Black points depict the IVW method was used to assess the causal effect, excluding single specific variant from the analysis. The red point denotes the inverse-variance weighted estimate using all SNPs.

**Abbreviations:** MR, Mendelian randomization; SNPs, Single nucleotide polymorphisms; IVW, Inverse-variance weighted.

**Figure** **S4 The causality of psoriasis on Her-2 positive breast cancer risk in European populations.** (A) Scatter plot. The slope of each line denotes the estimated effect of per mendelian randomization method. (B) Funnel plot. Vertical lines represent estimates with all SNPs. (C) Forest plot. The red points demonstrate the integrated estimates using all SNPs together, using IVW method. Horizontal lines represent 95% confidence intervals. (D) Leave-one-out analysis. Black points depict the IVW method was used to assess the causal effect, excluding single specific variant from the analysis. The red point denotes the inverse-variance weighted estimate using all SNPs.

**Abbreviations:** MR, Mendelian randomization; SNPs, Single nucleotide polymorphisms; IVW, Inverse-variance weighted.


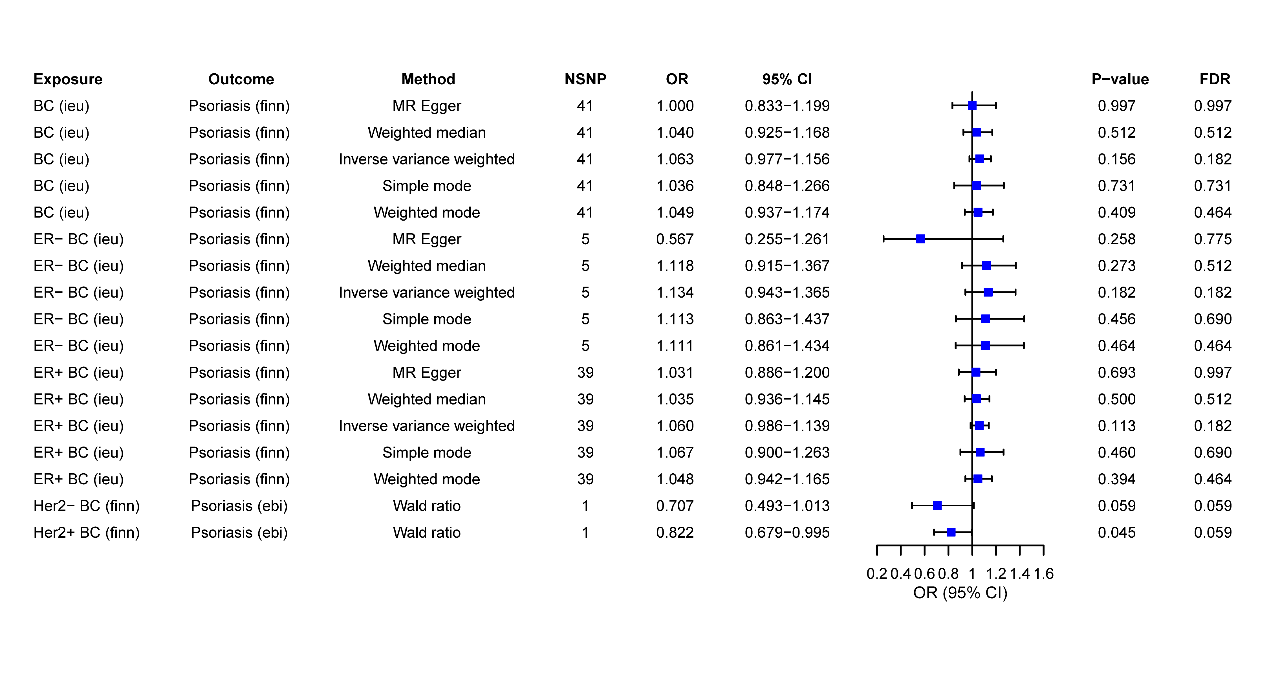


**Figure S5 The forest plots for breast cancer on psoriasis risk.** OR>1 indicates the positive correlation between a particular breast cancer and psoriasis. OR<1 indicates the Negative correlation between the two. The P<0.05 indicated statistical significance.

**Abbreviations:** MR, Mendelian randomization; ER-, ER-negative; ER+, ER-positive; SNP, Single nucleotide polymorphism; OR, Odds ratio; 95%CI, 95% confidence interval; NSNP, Number of sn p; FDR, False discovery rate; BC, Breast cancer.

**Figure S6 The causality of breast cancer on psoriasis risk in European populations.** (A) Scatter plot. The slope of each line denotes the estimated effect of per mendelian randomization method. (B) Funnel plot. Vertical lines represent estimates with all SNPs. (C) Forest plot. The red points demonstrate the integrated estimates using all SNPs together, using IVW method. Horizontal lines represent 95% confidence intervals. (D) Leave-one-out analysis. Black points depict the IVW method was used to assess the causal effect, excluding single specific variant from the analysis. The red point denotes the inverse-variance weighted estimate using all SNPs.

**Abbreviations:** MR, Mendelian randomization; SNPs, Single nucleotide polymorphisms; IVW, Inverse-variance weighted.


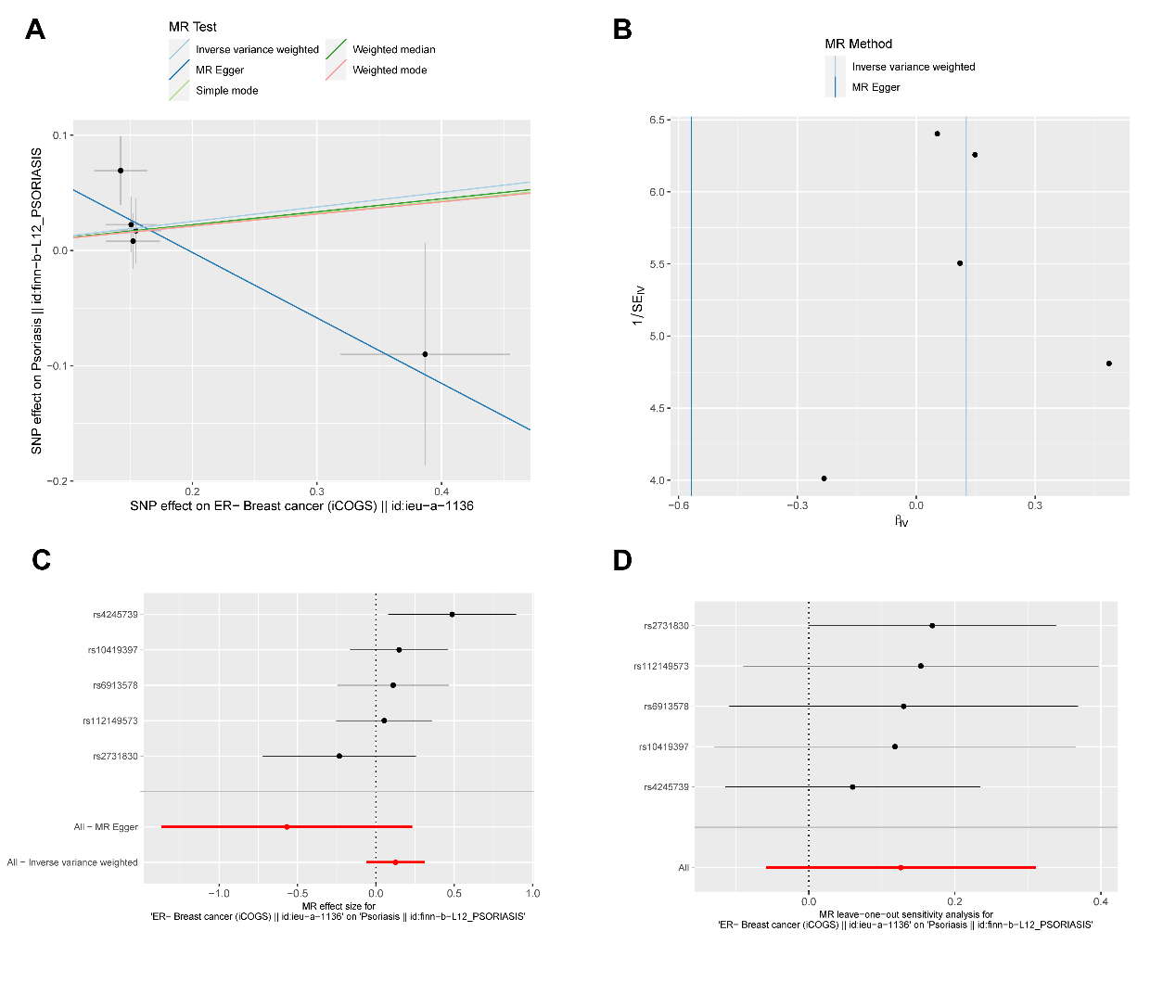


**Figure S7 The causality of ER negative breast cancer on psoriasis risk in European populations.** (A) Scatter plot. The slope of each line denotes the estimated effect of per mendelian randomization method. (B) Funnel plot. Vertical lines represent estimates with all SNPs. (C) Forest plot. The red points demonstrate the integrated estimates using all SNPs together, using IVW method. Horizontal lines represent 95% confidence intervals. (D) Leave-one-out analysis. Black points depict the IVW method was used to assess the causal effect, excluding single specific variant from the analysis. The red point denotes the inverse-variance weighted estimate using all SNPs.

**Abbreviations:** MR, Mendelian randomization; SNPs, Single nucleotide polymorphisms; IVW, Inverse-variance weighted.


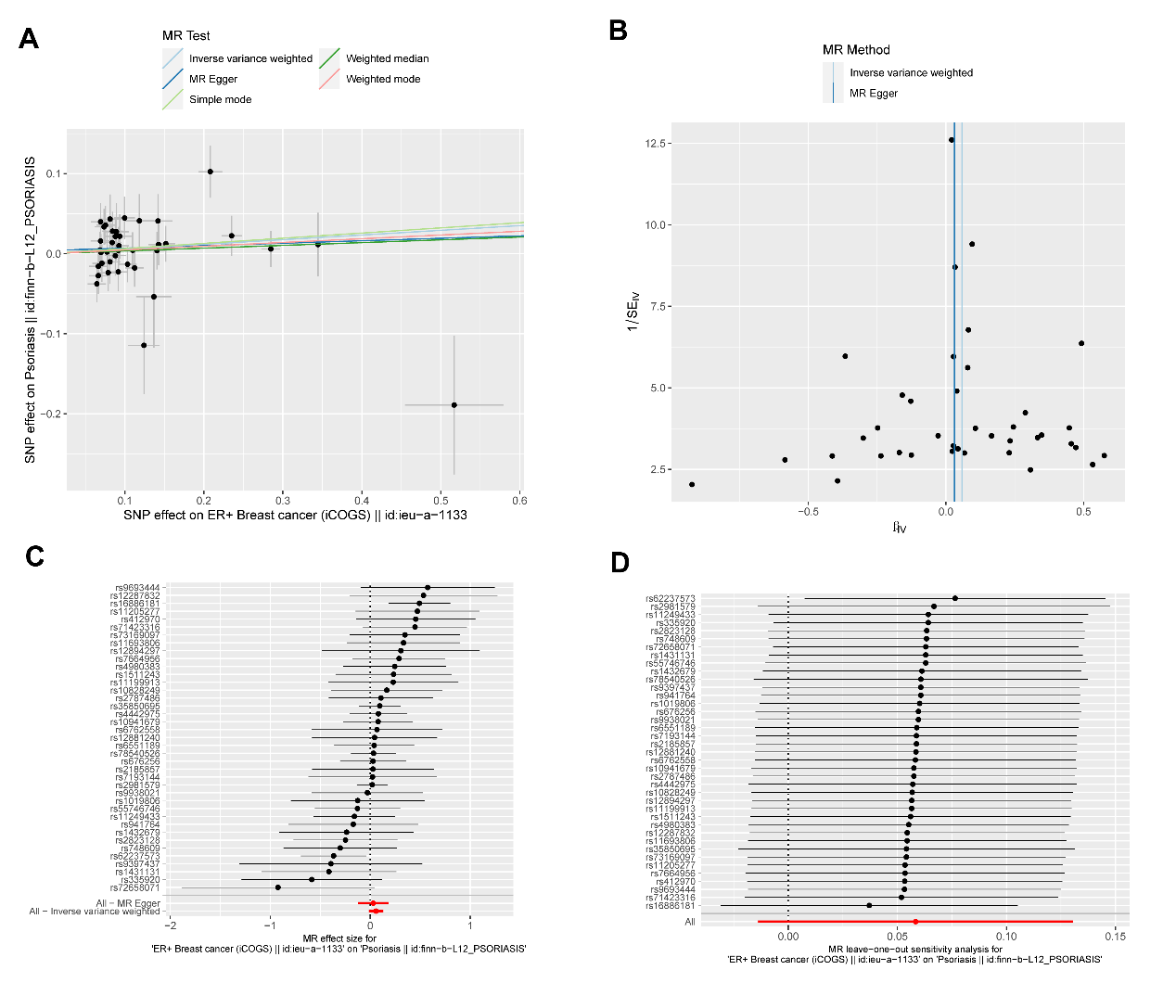


**Figure S8 The causality of ER positive breast cancer on psoriasis risk in European populations.** (A) Scatter plot. The slope of each line denotes the estimated effect of per mendelian randomization method. (B) Funnel plot. Vertical lines represent estimates with all SNPs. (C) Forest plot. The red points demonstrate the integrated estimates using all SNPs together, using IVW method. Horizontal lines represent 95% confidence intervals. (D) Leave-one-out analysis. Black points depict the IVW method was used to assess the causal effect, excluding single specific variant from the analysis. The red point denotes the inverse-variance weighted estimate using all SNPs.

**Abbreviations:** MR, Mendelian randomization; SNPs, Single nucleotide polymorphisms; IVW, Inverse-variance weighted.


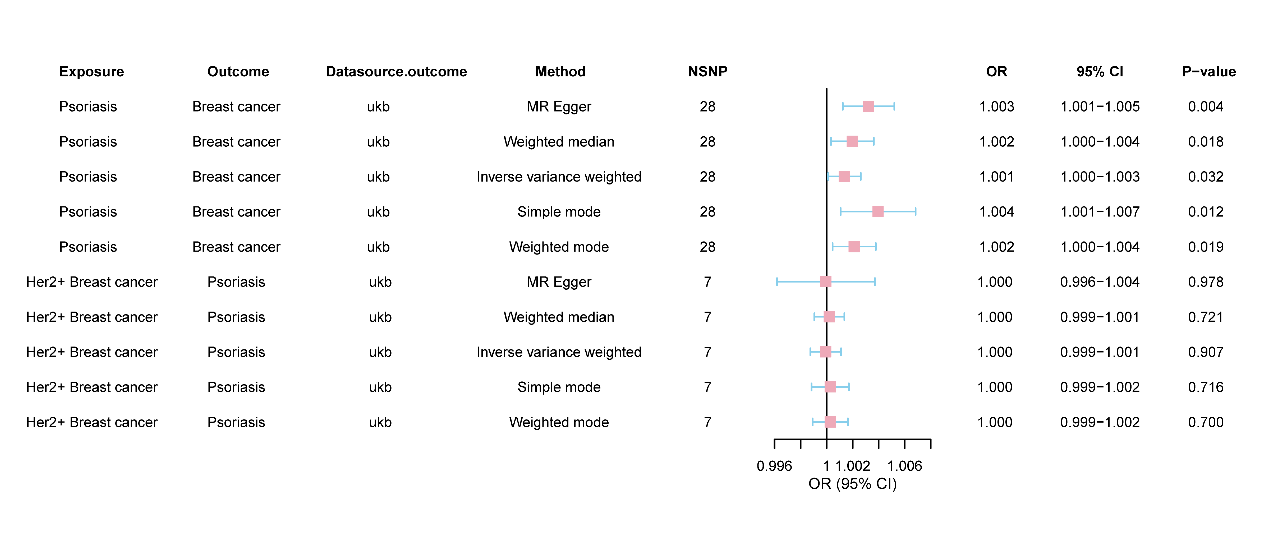


**Figure S9 Validation of statistically significant MR analysis results using the testing dataset.** OR>1 indicates the positive correlation between a particular breast cancer and psoriasis. OR<1 indicates the Negative correlation between the two. The P<0.05 indicated statistical significance.

**Abbreviations:** MR, Mendelian randomization; SNP, Single nucleotide polymorphism; OR, Odds ratio; 95%CI, 95% confidence interval; NSNP, Number of SNP.
